# Vegetation responses to past volcanic disturbances at the *Araucaria araucana* forest‐steppe ecotone in northern Patagonia

**DOI:** 10.1002/ece3.9362

**Published:** 2022-10-01

**Authors:** Ricardo Moreno‐Gonzalez

**Affiliations:** ^1^ Department of Palynology and Climate Dynamics University of Göttingen Göttingen Germany; ^2^ Institute of Conservation, Biodiversity and Territory Austral University of Chile Valdivia Chile; ^3^ Calahuala, cooperative for nature conservation Valdivia Chile

**Keywords:** disturbance regime, long‐term vegetation dynamics, Valdivian Temperate Rainforest hotspot, vegetation resilience, volcanic ecology

## Abstract

Volcanic eruptions play an important role in vegetation dynamics and its historical range of variability. However, large events are infrequent and eruptions with a significant imprint in today's vegetation occurred far in the past, limiting our understanding of ecological processes. Volcanoes in southern Andes have been active during the last 10 ka and support unique ecosystems such as the *Araucaria*–*Nothofagus* forest. *Araucaria* is an endangered species, with a fragmented distribution and well‐adapted to fire and volcanic disturbances. Yet, it was suggested that volcanism might have increased the fragmentation. Through the use of pollen and tephra analysis from a sedimentary record, this paleoecological study aims to provide an insight into the vegetation responses to past volcanic disturbances, to assess the role of volcanic disturbance on the vegetation dynamics and to determine if the current fragmentation has been caused by volcanism. Results show that during the last 9 kyr, 39 tephra falls buried the vegetation around Lake Relem, more frequently between 4 and 2 ka. The pollen percentage indicates that the vegetation changed after small tephra fall but seldom caused significant changes. However, the large eruption of Sollipulli volcano (~3 ka) changed the environmental conditions affecting severely the vegetation. *Ephedra* dominated the early successional stage, perhaps facilitating *Nothofagus* recovering after ~500 years. Slight increase of *Araucaria* and *Nothofagus obliqua*‐type pollen percentages suggests that forest resisted without permanent changes and recovered relatively fast after the large eruption, perhaps because of sparse biological legacies distributed in the landscape. In the study area, the relative stability of *Araucaria* pollen after several tephra fall suggests no change in its past distribution at the current forest‐steppe ecotone, thus not affecting its current conservation status. Perhaps, random factors, the colonization patterns of the high elevations in the Andes after deglaciation and topography might play a more important role than previously thought.

## Graphical abstract

Although past volcanic eruption might have affected the distribution of Araucaria' populations, thus its conservation status, I found no evidences that past tephra fall has impacted negatively the populations far from the volcanic source. Early succecional stages after severe tephra fall deposition were better explained by Ephedra' drought tolerance, dispersal, and regrowing mechanism, rather than the expected Nothofagus and Araucaria' shadow‐intolerant mechanism.

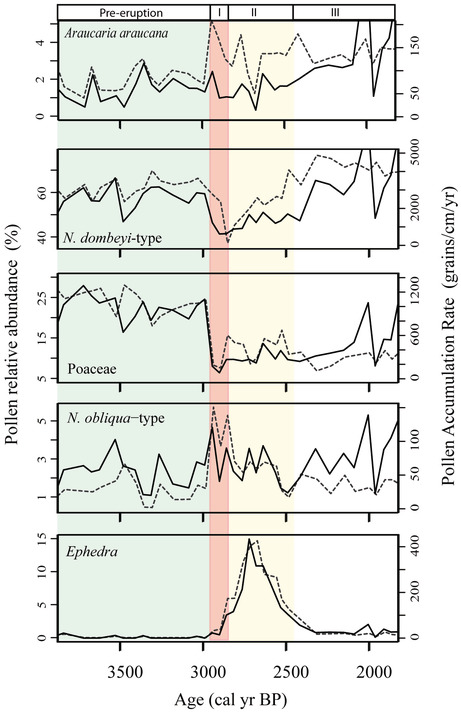

## 1 INTRODUCTION

Volcanic eruptions are important disturbance agents on Earth, and the impacts can trigger sudden and large environmental changes, particularly on the vegetation (Crisafulli & Dale, 2018). Large volcanic eruptions are infrequent, episodic, and stochastic events in the history of an ecosystem (Turner et al., 1998). Volcanism creates complex patterns and dynamic processes on the vegetation depending on the disturbance mechanism and the magnitude (e.g., Crisafulli & Dale, 2018; Foster et al., 1998). For example, after the eruption of Mount St. Helens in North America, the vegetation recovered rapidly after being buried by ashfall (Zobel & Antos, 2018), while literature suggests that the type and arrange of biological legacies in disturbed areas drove regeneration patterns and the rate of vegetation development after disturbance (del Moral & Grishin, 1999). Understanding the lasting effects of volcanism on vegetation responses like the resistance, recovery or the development of diversity patterns may help in designing nature management (Dale et al., 2005; Franklin et al., 2002) especially in highly diverse biogeographical areas influenced by volcanism and affected by current climate change. However, the study of the vegetation dynamics is challenging since active volcanoes are located in remote areas, the long‐term monitoring requires many resources, and the responses last too long to be monitored in human life span or had occurred far in the past (Swanson & Crisafulli, 2018) under different climate condition. For this end, well‐designed paleoecological studies, through the study of tephra layers and pollen in sedimentary records, can help to understand the consequences of past volcanic disturbances on vegetation responses or can be used as an analogue for future events (e.g., Payne & Egan, 2017).

Paleoecological studies in western Europe, North‐America, and New Zealand have attempted to illustrate past impacts of eruptions and aimed to contribute to disturbance ecology; however, results are not always consistent or show clear patterns. For example, palynological studies of large volcanic eruptions in Iceland and their effects on past climate showed contradictory or weak evidences that volcanoes disturbed the vegetation and past climate conditions in western Europe (Payne et al., 2013). Also in the Mt. Mazana, western North America, studies were conducted to infer the impacts on local and regional vegetation (e.g., Egan et al., 2016). The authors concluded eruptions did not trigger significant changes in terrestrial pollen taxa in a distant area, although noticeable increase in pollen dominance from Cupressaceae and *Tsuga heterophylla* following the eruption, while the aquatic taxa changed because of enrichment of nutrients. However, after the Taupo eruption (~1850 years BP), New Zealand, pollen evidences show that this eruption destroyed the surrounding forest, and up to 170 km east of the vent the vegetation suffered a variable degree of disturbance (Wilmshurst & McGlone, 1996). In many areas, the forest could not recover to its original conditions, and sites far from the crater covered by thin tephra layer were strongly impacted.

Active volcanoes during the Holocene are widely dispersed on the Earth, but one of the most active areas is located in the south‐eastern part of the Pacific (Stern, 2004). The subduction of the Nazca plate underneath the South American plate has triggered several large eruptions in the last 10 kyr in the Andes region (Fontijn et al., 2014). The southernmost section of the Andes supports unique forest ecosystems, characterized by a high endemism, which also has been influenced by these volcanoes eruptions affecting structure and functions in this Andean forest and surrounding vegetation (e.g., Veblen et al., 2016). As a result of the eruptions, the tree‐line location can be depressed (Daniels & Veblen, 2004; Veblen et al., 1977) or sustain uneven age forest in the landscape (Kitzberger, 2012). Volcanism was thought to have been responsible for keeping pioneer species such as *Nothofagus* species and *Araucaria araucana* dominant (Burns, 1991; Veblen & Ashton, 1978). The evidences showed that after the eruption of the Puyehue‐Cordon del Caulle Volcanic Complex (PCC) in 2011 *Nothofagus pumilio* was the principal species resprouting in zones buried by ~50 cm tephra, and the occurrence of several cohorts would correspond to past eruptions (Montiel et al., 2017). Eastward from PCC in 2011, the steppe vegetation was buried by <5 cm tephra and rhizomatous geophytes species such as *Poa* spp. and *Rumex acetosella* increased while therophytes disappeared (Ghermandi et al., 2015). The 2008‐eruption of Chaitén volcano was also studied. Swanson et al. (2013) described the early responses of the different types of disturbance (e.g., tephra falls, pyroclastic flows, and lahars) as similar to those that happened after the Mount St. Helens eruption, where each disturbance type impacted specific areas that created different patches in the landscape. Furthermore, Moreno‐Gonzalez et al. (2019) pointed out that in the direct blast zone of the 2008‐eruption of the Chaiten volcano the early vegetation establishment along the disturbance gradient was associated with elevation gradient, and that the regeneration depended on life‐traits strategies and the types of biological legacies (e.g., logs, branches, organic soil) remaining in the area.

Despite the volcanic characteristics and the unique vegetation, little is known about the role of volcanic disturbance on the vegetation dynamics in northern Patagonia, the rate of recovery, or the capability to resist disturbance, particularly in the *Araucaria araucana* forest. It was hypothesized that past volcanic eruptions might have influenced the genetic variability of *Araucaria araucana* populations but also affected negatively the population distribution and regeneration dynamics (e.g., Bekessy et al., 2002; Veblen et al., 2016). Yet the hypothesis is contradictory with the recognized morphological adaptation of this species (e.g., thick bark and flexible branches) to resist volcanic disturbance (Burns, 1991) that might have helped to persist in an angiosperm‐dominated ecosystems (Kershaw & Wagstaff, 2001). Despite *Araucaria*'s socio‐ecological importance and its current endangered conservation status, few long‐term vegetation reconstructions have been conducted so far in the *Araucaria* forest‐steppe ecotone. To contribute to the knowledge of vegetation dynamics and the volcanic disturbance regime, this work aims (1) to reconstruct the volcanic disturbance history in the *Araucaria* forest‐steppe ecotone, (2) to assess the vegetation responses to past volcanic disturbance, and (3) to evaluate if *Araucaria* distribution was influenced by volcanism at the current forest‐steppe ecotone affecting its conservation status. To this end, I reanalyzed a published pollen record (Moreno‐Gonzalez et al., 2021) to have an insight into the volcanic influence on the *Araucaria* forest dynamics. Due to the distance of the volcanic source of the forest‐steppe ecotone eastward of the Andes (~20–50 km), it is affected mostly by tephra fall that buried the vegetation. Tephra fall therefore is the main volcanic disturbance type around the study area. Following this assumption, I predict that the magnitude of vegetation responses should be related to the tephra thickness and that vegetation would be more resistant and/or recover faster to thin tephra fall than to thick tephra fall. In consequence, forest fragmentation should increase after thick tephra fall.

## 2 METHODS

**FIGURE 1:**
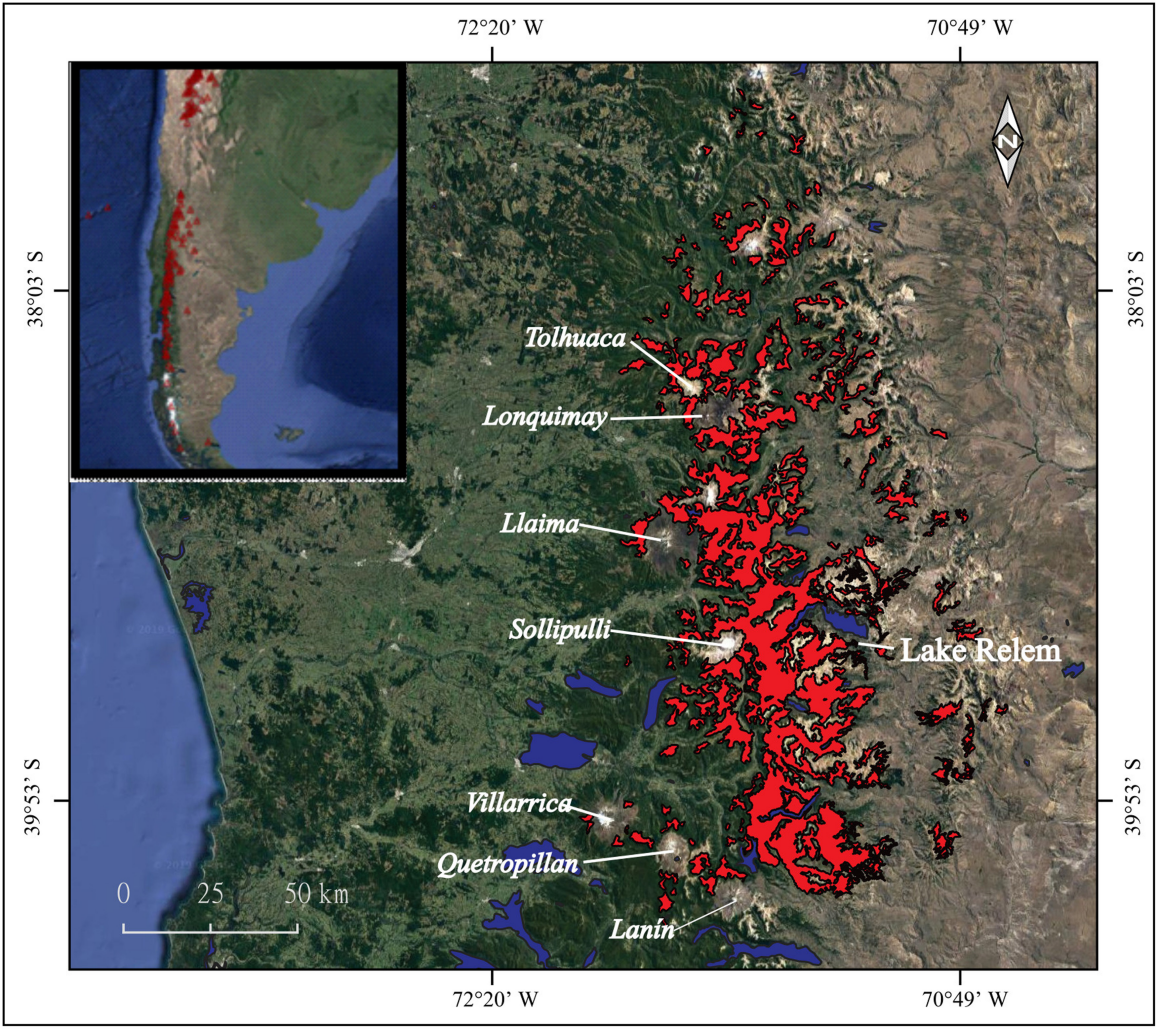
(a) Figure showing active volcanoes (red triangles) during the Holocene in southern South America (Global Volcanism Program, 2013). (b) Map of the study area representing the position of Lake Relem regarding the forest‐steppe ecotone, the location of volcanoes closes to Lake Relem, and the fragmented distribution of *Araucaria araucana* (red polygons). Base map source, Google (2020).

The study area is located in the current *Araucaria* forest‐steppe ecotone around 39° S, in northern Patagonia (Figure 1). Vegetation history was reconstructed from a sediment core obtained from Lake Relem (38°58′39″S; 71°4′51″W; 1268 m a.s.l.). Lake Relem is a shallow and small lake (~2.5 m depth, ~1 ha, respectively), without river inflow or outflow. The climate in the region is temperate with oceanic influence. The Pacific air masses arriving with westerly winds create a sharp rain‐shadow effect discharging most of the precipitation on the western section of the Andes (Mundo et al., 2013). Climatic conditions when interacting with local topography and natural disturbances generate complex vegetation patterns and plants associations (Kitzberger, 2012; Roig, 1998)*. Araucaria* forest occurs mainly above 1000 m elevation, normally reaching the tree‐line at 1700 m a.s.l. (Gonzalez et al., 2006) showing a strong fragmentation (Figure 1). Further details on climate and vegetation description, as well as a full description of the sediment analysis, pollen analysis, and the chronology can be found in Moreno‐Gonzalez (2020) and Moreno‐Gonzalez et al. (2021). In brief, the sediment core was dated with 7 AMS Radiocarbon dates and then the chronology was made with smooth spline (0.1 spar) at 95% confidence. At least 400 pollen grains were counted in each of the 176 pollen samples. The pollen samples were obtained every 2–4 cm, most of them in the following first 1–4 cm above the tephra layers. Time resolution per sample varied between ~−20 year/cm above the So‐A tephra layer and ~40 year/cm below the So‐A tephra layer.

### 2.1 Volcanic setting and eruptive history

The study area lies in the Southern Volcanic Zone of the Andes (SVZ). The SVZ is a result of the subduction of the Nazca plate beneath the continental South American plate and is extended between 33°S and 46°S (Gilbert et al., 2014). In a radius of 100 km around Lake Relem, there are seven volcanoes that have been active during the Holocene (Figure 1). Fontijn et al. (2014) list all eruptions recorded so far to the SVZ, which are summarized in Table 1. The Lonquimay volcano has had more regular eruptions of considerable magnitude (Volcanic Eruption Index ≥ 3) while other volcanoes have erupted every 10 years (e.g., Villarrica volcano) or have erupted at least once during the last 10 k years (Fontijn et al., 2014). The Sollipulli–Alpehue eruption (hereafter the So‐A eruption) was one of the largest in the recent past in northern Patagonia, dated back to 2951 cal year BP (Naranjo et al., 1993). Lake Relem is located about 40 km from the Sollipulli volcano within the tephra isopach of 2 m of the So‐A eruption (Fontijn et al., 2016).

**TABLE 1:** Location of potential source volcanoes close to Lake Relem, their frequency during the Holocene and amount of eruptions of considerable magnitude (VEI: Volcanic Eruption Index; further details of dates and uncertainty, chemical composition, references, for each eruption, are found in Fontijn et al., 2014).

Volcano name	Distance and direction from Lake Relem	Total eruptions last 10 ka	Eruptions VEI ≥ 3
Tolhuaca	88 km; 326°NNW	1	1
Lonquimay^a^	80 km; 326°NNW	22	18
Llaima	64 km; 299°WWN	55	3
Sollipulli	38 km; 270°W	2	2
Villarica	89 km; 236°WS	150^b^	10
Quetropillan	80 km; 223°WS	3	1
Lanin	81 km; 205°WSS	5	4

^a^ In Lonquimay, VEI is not provided by Fontijn et al. (2014), but the composition of tephras (mainly dacite) probably ejected about 0.01 km^3^ of tephra, equivalent to VEI ≥ 3 (Gilbert et al., 2014).

^b^ Some of the eruptions the dates are uncertain. Most of them occurred in the last 500 years and have been recorded in historical documents.

### 2.2 Data analysis

For this study, I used pollen percentage and pollen accumulation rate (PAR) data from terrestrial taxa following standard method (Bennett & Willis, 2001). Calculation and methodology were further described in Moreno‐Gonzalez (2020). A stratigraphically constrained cluster analysis (CONISS) of the pollen samples (Grimm, 1987) was conducted based on Euclidean distances and calculated the amount of significant cluster zones by broken stick method (Bennett, 1996). To assess pollen diversity changes, individual rarefaction analysis is a powerful tool (Birks & Line, 1992). Using rarefaction analysis, we estimated the palynological richness at the minimum terrestrial pollen count of 400 pollen grains (E(*T*_400_)), the pollen diversity estimated at E(*T*_10_), and pollen evenness was calculated as the ratio E(*T*_10_)/E(*T*_400_) (Matthias et al., 2015). Compositional trend of the terrestrial taxa was explored through principal component analysis. The percentage was square root transformed and centralized. Furthermore, we fitted a principal curve (PC) to the compositional data. The starting point was based on the age of the samples, and the curve was fitted through a smooth‐spline method with complexity of 5. To estimate the rate‐of‐change (RoC), we interpolated the pollen samples at a regular time interval of 50 years with smooth spline and then with the Euclidean distances as a dissimilarity coefficient (Bennett & Humphry, 1995).

To reconstruct the volcanic disturbance regime, I made use of every tephra layer >0.5 cm thick, which was considered arbitrarily as a clearly deposited layer and not mixed with the sediment. Here I considered each of these tephra layers as a single and independent disturbance event—since tephra fall is limited to a short time—and the tephra thickness is considered as a measure of the magnitude of the disturbance caused by the tephra burying the vegetation in the surrounding of the studied lake (regardless of the eruption magnitude, distance from the crater, wind direction, among other factors that could cause differences in the thickness of the deposited layer in a particular place). Furthermore, each tephra was coded as a quantitative explanatory variable and modeled as a simple exponential decay process (Lotter & Birks, 1993). This model is a simple but robust equation (*x* ^−*αt*^), where *x* is the value for the ash (arbitrarily set to 100 by the authors), α is the decay coefficient equal to −0.5, and *t* is the sample depth (=time). Also, the authors arbitrarily assigned a value before tephra deposition of 0. In this article, I preferred to describe the magnitude of the event of each tephra by giving the ash‐value corresponding to the tephra thickness in centimeters. In doing so, I aim to describe the magnitude of the eruptions. Unlike the record of Lotter and Birks (1993), in sedimentary records from Patagonian the occurrence of multiple tephra is quite normal; therefore, the as value below a tephra layer corresponded to 0 only in some cases. The frequency of volcanic events was calculated as the sum of events over 1000 years and then was modeled with smooth‐spline method (spar = 0.7). Both variables, frequency and magnitude, were later used as an explanatory variable to constrain the pollen samples in a multivariate analysis (Redundant Analysis) assessing vegetation responses to volcanic disturbance regime. All statistical analyses were conducted through RStudio 3.3.1 (RStudio Team, 2016), vegan‐package 2.4‐2 (Oksanen et al., 2017), and Rioja 0.9‐15 (Juggins, 2015).

## 3 RESULTS

### 3.1 Reconstruction of the volcanic regime

**FIGURE 2:**
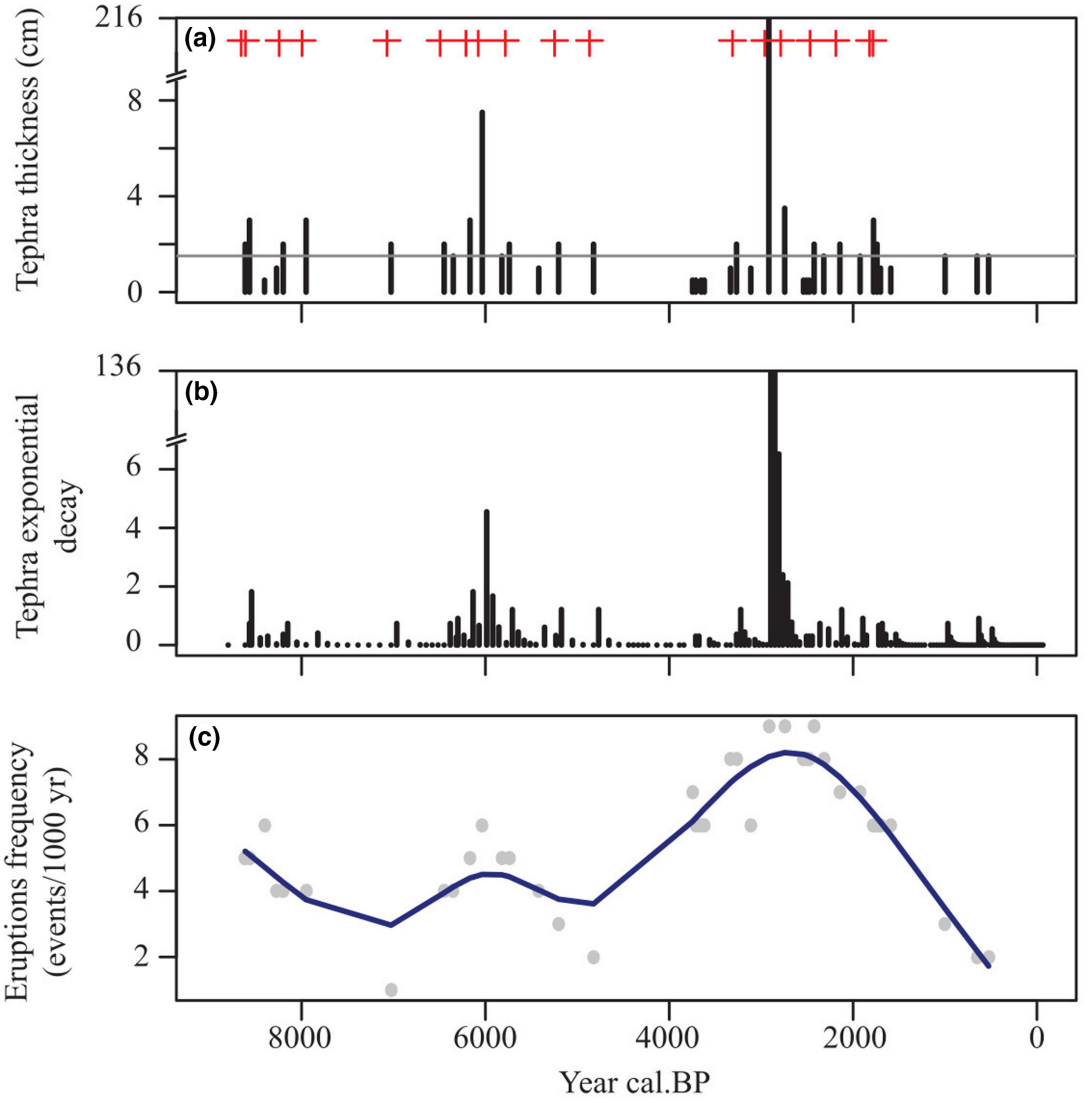
Volcanic eruption regime. (a) Indicates the thick of tephra layers deposited into the Lake Relem. Gray horizontal line shows arbitrary threshold of >1.5 cm to determine relevant eruptions (red crosses). (b) Tephra thickness modeled as the exponential decay after eruption. (c) Eruption frequency every 1000 years. Gray points indicate the sum of frequency every 1000 years. Trend of frequency was obtained by smooth‐spline function (blue line) with spar = 0.7.

The volcanic history and its disturbance regime were reconstructed from the number and thickness of tephra layers in the sediment of Lake Relem (Figure 2). The sediment core registers 39 tephra layers, well‐defined >0.5 cm thick. Most of them are between 0.5 and 7.5 cm thick (Figure 2a) but only 18 are >1.5 cm thick, what could be considered important, representing large eruptions (crosses in Figure 2a). The tephra layer corresponding to the Alpehue eruption from the Sollipulli volcano (Naranjo et al., 1993) is the biggest one, with 216 cm thickness. The tephra value, modeled as an exponential decay, shows similar patterns regarding the tephra thickness but indicates a slight effect regarding to the eruption magnitude while missing some peaks above the threshold (Figure 2b). Considering all the tephra layers, the overall volcanic disturbance frequency is relatively high (Figure 2c). The disturbance frequency indicates that around seven eruptions had occurred every 1000 years between 4 and 1.8 ka. Before 4 ka volcanic frequency was <6 events/1000 years, with periods without volcanic disturbance around 7 and 5 ka. For the last 1.5 ka to the present, few tephra layers are recorded, but none deposited over 1.5 cm of tephra into the lake sediment.

### 3.2 Vegetation responses to volcanic events

**FIGURE 3:**
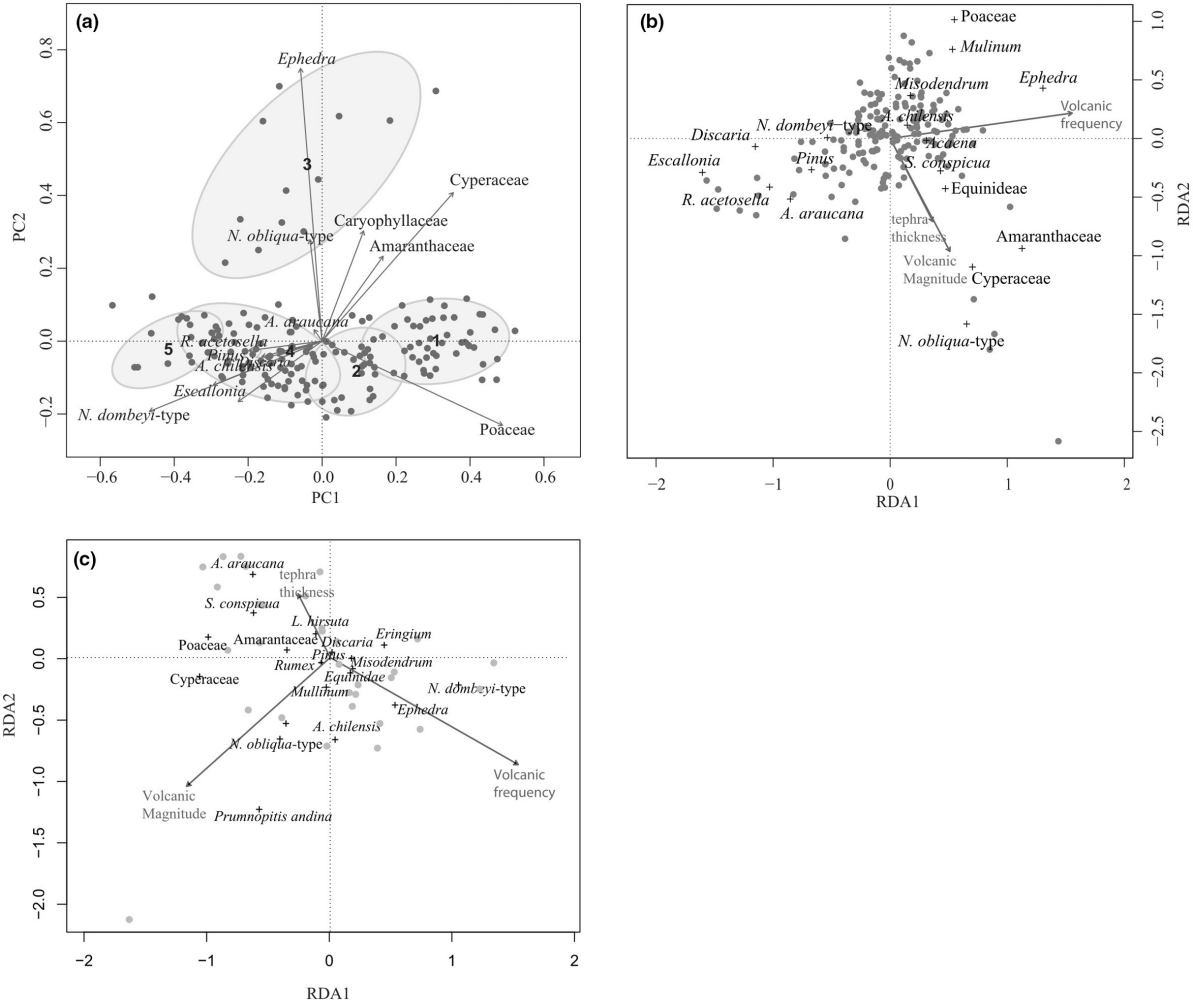
(a) Ordination diagrams of pollen composition for Lake Relem. Ellipses indicate the different pollen zones. Fifth group topmost samples indicating the more recent time. (b) Pollen composition from all samples constrained by volcanic parameter describing the disturbance regime: Volcanic magnitude (*p* = .039), volcanic frequency (*p* = .001), and tephra thickness (*p* = .076). (c) Pollen composition, without samples of zone 3 influenced by Sollipulli‐Alpehue eruption, constrained by volcanic parameter describing the disturbance regime: Volcanic magnitude (*p* = .139), volcanic frequency (*p* = .103), and tephra thickness (*p* = .824).

A full description of the vegetation history was published in Moreno‐Gonzalez (2020). It encompasses the last 9 kyr, and it is characterized by an overall vegetation transition that occurred ~4.5 ka from an open *Nothofagus dombeyi*‐type woodland‐grassland vegetation that transitions to a more closed forest at 4.5 ka (Appendix 1), likely as a result of a change in precipitation regime rather than fire (Moreno‐Gonzalez et al., 2021). Likewise, in the unconstrained ordination diagram (Figure 3a) the compositional trends represent the gradual vegetation change. The first component explains 78% of the total variance and would be interpreted as the long‐term shift from a vegetation mosaic of mixed steppe taxa dominance (e.g., Poaceae) with open woodland to a forest with the dominance of *N. dombeyi*‐type. Poaceae, *Mulinum*, and Cyperaceae, among others, are more abundant in the zone 1, on the left side of the ordination diagram. The second component explains 11% of the variance and splits the overall trend where *Ephedra* is dominant. *Ephedra*'s abundance in the pollen record rose after the So‐A eruption (Figure 3, Appendix 1); therefore, the second axis is mostly related to the vegetation response to the So‐A (Figure 3a). Volcanic eruptions had a significant influence on vegetation composition (Figure 3b) explaining 20% of the data. The explanatory variables, volcanic frequency and magnitude, had a significant influence on the vegetation, too. In the multivariate analysis, *Araucaria* shows a weak negative relation to volcanic frequency, while somewhat related with volcanic magnitude, suggesting that *Araucaria* increased its abundance following the eruptions, likely influenced by intense eruptions. However, further multivariate analysis excluding samples after the So‐A eruption (Figure 3c) indicates that the other small tephra layers had no significant influence in the pollen composition.

**FIGURE 4:**
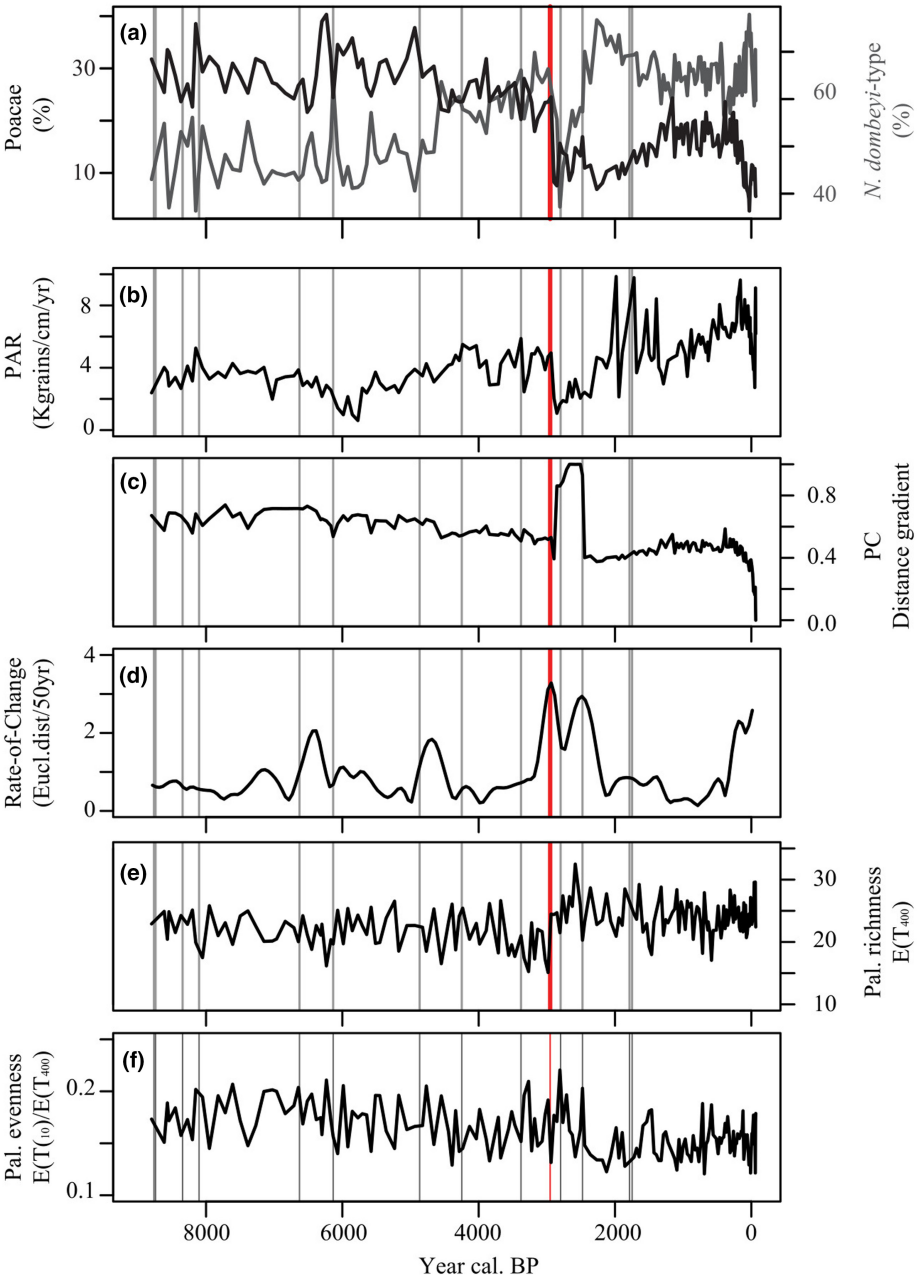
Vegetation responses to volcanic eruptions registered in the sediment record from Lake Relem. (a) Pollen relative abundance of selected dominant *Nothofagus dombeyi‐*type (dark gray curve) and Poaceae (black curve). (b) All pollen taxa pollen accumulation rate used as a proxy for vegetation biomass changes. (c) Principal curve and (d) the rate of change showing significant compositional changes along the time. (e) Palynological richness E(*T*_400_)) and (f) palynological evenness E(*T*_10_)/E(*T*_400_) comparing diversity changes after volcanic disturbances. Red vertical line indicates the Sollipulli‐Alpehue eruption; gray vertical lines indicate other tephra deposited >1.5 cm thick into the lake.

Figure 4 shows several indicators of the vegetation in relation with the So‐A eruption (vertical red line) and other unknown tephra between 1.5 and 10 cm thick (vertical gray lines) influencing the study site. Tephra fall that marked a disturbance event normally caused a decrease in Poaceae and an increase in *N. dombeyi*‐type pollen percentages (Figure 4a). The decrease is not proportional to tephra thickness, but it is more evident before 4.5 ka, when vegetation was richer in Poaceae and other grasses. Only So‐A eruption depressed the abundance of both co‐dominant taxa, while the few events after So‐A do not seem to have affected any of the pollen taxa. Along the record the PAR of all taxa is variable (Figure 4b), where, although further calibrations are needed, PAR could be interpreted as an indicator of vegetation biomass (Seppä et al., 2009). Volcanic events normally caused small decreases in PAR between 100 and 500 grains cm^−2^ year^−1^, while after So‐A eruption, PAR decreased by over 2000 grains cm^−2^ year^−1^. Other drops in PAR can be related to sedimentary processes or other disturbances. The PC fitted after 21 iterations. Its variation in the distance gradient units shows some sensitivity of the vegetation composition to volcanic disturbances; however, there are only few significant changes (Figure 4c). Most relevant changes occurred at the time of So‐A, but the curve also suggests that pollen composition was relatively stable through the time. On the contrary, peaks depicted by the RoC were not sensitive to volcanic events, except for So‐A (Figure 4d). The result shows that the minor changes after thinner tephra deposition did not have long‐lasting effects on the vegetation and could recover rapidly after the events. Changes in palynological richness are variable and are not always clearly related to volcanic disturbance (Figure 4e). In response to volcanic disturbance, palynological richness can increase or decrease. In particular, before the So‐A eruption, palynological richness showed lower values coinciding with the expansion of *N. dombeyi*‐type in the area; but, interestingly after So‐A eruption, palynological richness increased. Perhaps vegetation was not completely destroyed, with some biological legacies that regrowth after buried (e.g., ephemeral Cyperaceae) and surviving individual remained in sheltered areas. As the dominant taxa decreased, palynological evenness increased (Figure 4e), a pattern that normally occurred after other small volcanic eruptions in this study.

## 4 DISCUSSION

After volcanic disturbance episodes, the pollen richness and evenness, as well as PAR, do not indicate a unique pattern of decreasing biodiversity and biomass. In general, volcanic eruptions were considered creating barren areas, starting a new (primary) ecological succession likely dominated by pioneer and shadow‐intolerant species, characterized by low plant biomass and diversity. Contrary to this classical model, the findings presented here showed a more complex dynamic in the *Araucaria* forest‐steppe ecotone, what may be controlled by many interacting factors. Recent advances in volcanic ecology (Crisafulli et al., 2015) point that the vegetation resistance and the rate of recovery result from factors such as climatic conditions, type of the impact and topography (del Moral & Grishin, 1999), plants traits (Antos & Zobel, 1986), and biological legacies (e.g., Dale et al., 2005). Moreover, those operating factors can vary in time and spatial extension from one eruptive event to another. In the following paragraph, I attempt to contextualize those patterns occurring around Lake Relem.

**FIGURE 5:**
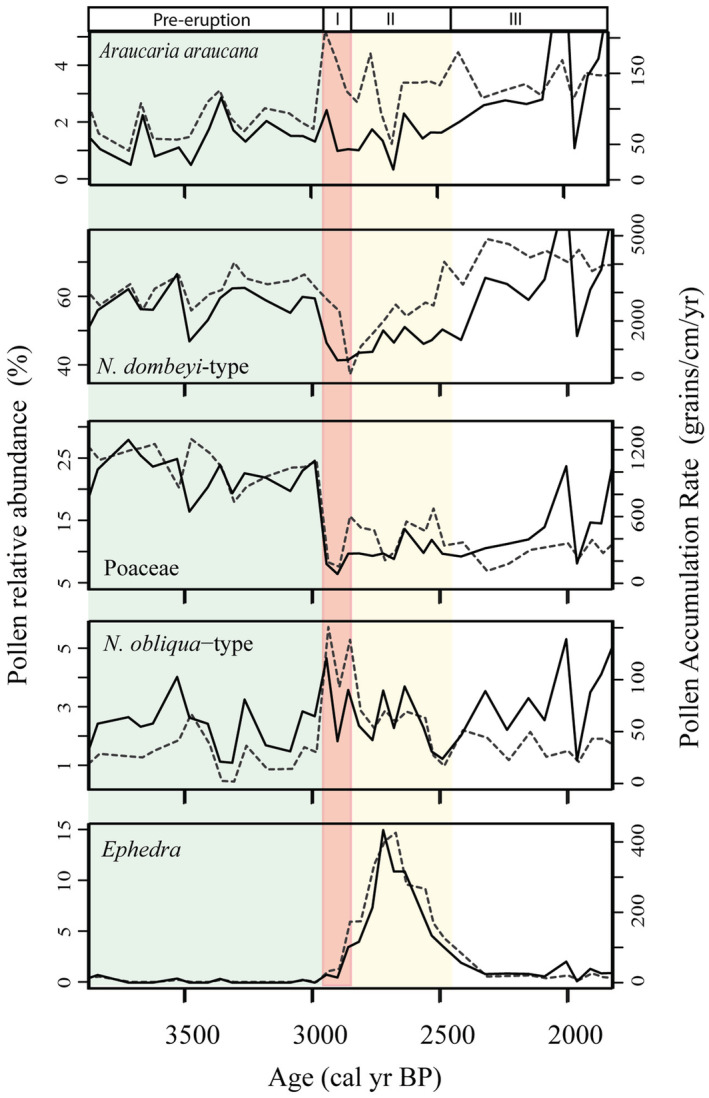
Successional patterns caused by the Sollipulli‐Alpehue eruption. Primary *y*‐axis shows pollen relative abundance (dotted curve) and secondary *y*‐axis shows the pollen accumulation rate (continuous curve) of selected taxa. Small boxes at the top indicate successional phases. Pre‐eruption is represented in green. Phase I (reddish area) indicates the “collapse” of the vegetation. Phase II (yellowish area), colonization and expansion into the disturbed area. Phase III, reorganization and recovery of original conditions. Note that periods of time are not exact and are used only with schematic purpose.

Although Cyperaceae (a common element of the steppe grassland) showed the first ephemeral increase after the So‐A eruption, *Ephedra* showed an unexpected increase dominating the early successional stage after the So‐A eruption and not the expected *Nothofagus* species and *Araucaria* which are considered pioneer trees in the region (Figure 5). Little is known about *Ephedra*'s ecology and its palaeoecological significance in Patagonia, but in a study ~200 km southward from Lake Relem, it was found that *Ephedra* might have a nursery effect on *Nothofagus* and *Austrocedrus* after fire disturbance (e.g., Raffaele & Veblen, 1998) although *Ephedra* is not the most abundant species after disturbances. Modern pollen samples show that *Ephedra* averages 2.7% in Patagonia with maxima of 32.4% in some areas, but close to Lake Relem percentages are up to 20% (Paez et al., 2001). *Ephedra*'s pollen grains have been known to disperse several kilometers far from the source area, but with low abundance (Maher, 1964). The pollen abundance of *Ephedra* before So‐A eruption suggests a persistent presence but was always low; thus, the increase in pollen percentage and PAR following the So‐A eruption should indicate a very local presence. Perhaps the species were suppressed or co‐existing with other species before So‐A eruption and the following increase as a result of the So‐A eruption that facilitates its expansion under certain conditions. For instance, at Mallín Paso del Arco (Heusser et al., 1988), a record within the 2 m isopach zone of the So‐A, a slight increase of *Ephedra* is seen around 3 ka, above a thick tephra layer at the core bottom. However, other sites do not show an increase in *Ephedra* related to tephra (Dickson et al., 2020; Fontana & Giesecke, 2017) and, to my knowledge, none of the palynological records in northern Patagonia show a significant increase of *Ephedra* after an eruption nor have been reported following recent eruptions.

Climatic condition may play a role in the environmental responses by determining the vegetation composition and structure, which in turn influence potential colonizer species and the rate of revegetation after disturbance. For example, before 4.5 ka when precipitation was low (Jenny et al., 2003; Lamy et al., 2001), grasses were more abundant (Figure 4). Thin tephra fall left some impact on the vegetation dominated by grasses (Figure 4b), but recovering fast enough to not register a peak in the RoC analysis (Figure 4c). After So‐A, revegetation started shortly after with *Ephedra* whilst forest developed later. This eruption evidenced a strong imprint in vegetation composition, but due to relatively stable climatic conditions in a millennial scale it returned close to its original state after approximately 500 years (Figures 4 and 5). In comparison, after the Taupo eruption in New Zealand, the vegetation returned to its original condition after about 120–250 years (Wilmshurst & McGlone, 1996). Later, when precipitation augmented in Patagonia, it promoted the development of *Nothofagus* species in the area. Then, the vegetation was less sensitive to small tephra deposition that might cause short‐term effects—hardly detectable in the sediment—or was not disturbed significantly. Future climate trends in northern Patagonia could be characterized by more frequent and intensive drought (Garreaud, 2019), what might drive current degraded forest fragments to open woodland combined with widespread grassland as occurred in the past. Although uncertain, a huge eruption, like the So‐A, would probably cause catastrophic ecological impacts on a vegetation subjected to persistent dry climatic condition.

Plant traits play also an important role in resisting tephra deposition and may determine the species performance. For example, Antos and Zobel (1986) found that herbs could not resist being buried by ~15 cm, while the early establishment was dominated by the shadow tolerant trees and shrubs under the undisturbed canopy. Regarding the So‐A eruption, the early establishment of *Ephedra* was likely because of the taxon's special adaptation to poor soil, sprouting shoots, and the capacity to resist arid and cold weather conditions (Luebert & Pliscoff, 2006). Sprouting from roots seems to be a common mechanism of plants in response to buried disturbance by tephra fall. After the Taupo eruption (~1850 BP), the spores of *Pteridium*, a fern species with rhizome root system, were found increasing in several records in a wide area buried by tephra fall, thus suggesting the species spread after resisting the impacts of the eruption (Wilmshurst & McGlone, 1996). The same occurred with the fern *Lophosoria quadripinnata* after the 2008‐eruption of Chaitén volcano. The species possess also a rhizome root system and normally grows under the forest canopy in the low elevation of the temperate forest. Close to the blast zone, *Lophosoria* was one of the first species to grow 1 year after the eruption in low and mid‐elevation zones (Moreno‐Gonzalez et al., 2019). Remarkably, after repeated past eruptions in the areas close to Chaitén Volcano, *Lophosoria* responded positively shortly after the eruptions (Henríquez et al., 2015). Furthermore, the following expansion of *Ephedra* in the area (Figure 5, Phase II) can be related to its fleshy‐fruit and dispersal mechanism, principally through birds and rodents (Loera et al., 2015). Probably *Ephedra* was the major food supply for birds and rodents in a wide area devastated after So‐A eruption. A similar mechanism of plant dispersal was suggested after the Taupo eruption, where the pollen of some fleshy‐fruits taxa was found (Wilmshurst & McGlone, 1996), and in the last eruption of the Chaitén volcano, the species with fleshy‐fruits were also found playing an important role in the early vegetation establishment (Moreno‐Gonzalez et al., 2019).

Trees are expected to be less affected by thin tephra fall, particularly *Araucaria* and *Nothofagus* species which are considered pioneer species after volcanic disturbances (Veblen, 1982). After the recent eruption of Puyehue, *Nothofagus pumilio* species was found resprouting in areas buried by tephra (Montiel et al.,  2017). However, in the blast zone of the last eruption from Chaitén Volcano, none *Nothofagus* seedling were found 1 year after the eruption (Moreno‐Gonzalez et al., 2019). In palynological records, the abundance of *N. dombeyi* type is always variable, and few studies have assessed directly the responses to volcanic events. Álvarez‐Barra et al. (2020) demonstrated that well‐developed forests were not affected in the long term by tephra fall smaller than 20 cm. However, after the large eruption of So‐A, the pollen abundance of *N. dombeyi*‐type was strongly affected (this study and Dickson et al., 2020). Indeed, the data presented here show that *Nothofagus* species did not behave as pioneer and expanded into the area only after 500 years in advanced successional stages following the decrease of *Ephedra* (Figure 5, phase III).

Multivariate analyses indicated *Araucaria* was not affected by any of the thin tephra fall. It is largely documented that the thick bark, flexible branches and smooth leaves allow the species to resist moderate disturbance (Burns, 1991; Veblen et al., 2005). Hence, it is possible that thin tephra fall deposited in the crown of the trees did not cause significant physical damage to the entire population. If chemical change to soil occurred after tephra deposition, physiological harm such as decreasing tree‐ring growth might not last for a long time (Tognetti et al., 2012); therefore, it is unlikely to produce a significant change in pollen abundance from sedimentary records. Since *Araucaria* did not significantly change in pollen percentage and PAR after the So‐A eruption (the largest eruption in the area for the last 9 ka), it is unlikely that volcanism affected negatively the distribution of the populations nor increased the forest fragmentation in areas distant from a volcanic source as it suggested by Bekessy et al. (2002). A recent pollen record from Lake Cilantro (Dickson et al., 2020) shows similar patterns to those observed in Lake Relem. The authors recorded neither changes in pollen relative abundance after small tephra layers nor changes in PAR after So‐A eruption. Instead, Dickson et al. (2020), Moreno‐Gonzalez et al. (2021), and Nanavati et al. (2020) showed that pollen abundance in areas near the current ecotone has been slightly increasing for the last ~4 ka despite the influence of several volcanic eruptions. Further palaeoecological studies need to be done to understand the role of past volcanic eruptions in determining current diversity patterns in *Araucaria* forest and that can be used in the conservation of certain threatened species in the Valdivian Temperate Rainforest hotspot.

## AUTHOR CONTRIBUTION

**Ricardo Moreno‐Gonzalez:** Formal analysis (equal); investigation (equal); methodology (equal); writing – original draft (equal); writing – review and editing (equal).

## CONFLICT OF INTEREST

None conflict of interest.

### OPEN RESEARCH BADGES

The data for this research are stored at the PANGAEA in the following link: https://doi.org/10.1594/PANGAEA.923741.

## Data Availability

Moreno‐Gonzalez et al. (2020): Pollen and macro‐charcoal analysis of Lake Relem sediment core, northern Patagonia. https://doi.org/10.1594/PANGAEA.923741.

## APPENDIX 1

Short pollen diagram of selected taxa. Full pollen diagram is given in Moreno‐Gonzalez (2020).
